# Perceived stress and problematic micro-short viewing among Chinese university students: mediating effects of state boredom and moderating effects of loneliness

**DOI:** 10.3389/fpsyg.2026.1808559

**Published:** 2026-09-10

**Authors:** Yun Zhou, Hao Yuan, Man Chen, Cheng Wei, Changjie Fan, Xuyan Wang, Long Chen, Guoqiang Lu, Yisong Yang

**Affiliations:** 1 School of Art, Chengdu Jincheng College, Chengdu, China; 2 Faculty of Psychology, Beijing Normal University, Beijing, China; 3 College of Education, Chengdu College of Arts and Sciences, Chengdu, China

**Keywords:** college students, loneliness, perceived stress, problematic viewing of micro-short dramas, state boredom

## Abstract

**Background:**

Problematic viewing of micro-short dramas has emerged as a significant public health issue among Chinese university students. Based on General Strain Theory and the I-PACE model, this study examined whether state boredom mediates the relationship between perceived stress and problematic viewing, and whether loneliness moderates this mediation pathway.

**Methods:**

A cross-sectional study was conducted with 781 Chinese college students (*M* = 21.10 ± 1.56 years; 50.83% female). Participants completed the Perceived Stress Scale-10 (PSS-10), Multidimensional State Boredom Scale (MSBS), UCLA Loneliness Scale Version 3 (ULS-20), and a revised Problematic Micro-Short Drama Viewing Questionnaire. PROCESS Model 7 with 5,000 bootstrap samples was used to test the moderated mediation model.

**Results:**

Perceived stress was significantly positively correlated with problematic viewing (*rs* = 0.352, *p* < 0.01). State boredom showed a significant indirect association linking perceived stress with problematic viewing, consistent with a partial mediation pattern in the hypothesized model (indirect effect = 0.136, 95% CI [0.080, 0.199], accounting for 15.03% of the total effect). Loneliness significantly moderated the stress-boredom pathway (*β* = 0.138, *p* < 0.001): the effect was significant at high loneliness levels (*β* = 0.327, *p* < 0.001) but not at low levels (*β* = 0.051, *p* = 0.130). The indirect effect was significant for high loneliness (Effect = 0.114, 95% CI [0.085, 0.145]) but not for low loneliness.

**Conclusion:**

These findings suggest that perceived stress is related to problematic micro-short drama viewing through state boredom, with loneliness acting as a “risk amplifier” in the stress-boredom-viewing pathway. Interventions targeting both stress management and social connection may effectively prevent problematic viewing among at-risk students.

## 1 Introduction

Micro-short dramas are a new, immersive entertainment form thanks to mobile internet, big data, and cloud computing. Many in film and television are producing short dramas of a few seconds to 15 min, with clear themes and complete stories. High-density storytelling, concise pacing, and algorithm-driven recommendations quickly attracted many fans. Low production costs and wide appeal make this format likely to spread globally. Data from iMedia Research show the Chinese micro-short drama market is booming. Many dramas are now translated and viewed overseas. In 2023, the market reached 37.39 billion yuan, up 267.65% year over year. By June 2024, users of platforms like Douyin and Hongguo exceeded 576 million; over 70% are young people, making them the core audience. However, rapid market expansion has led to public health concerns. Weak new media regulation, content sameness, and intense algorithm-based recommendation encourage problematic viewing, making it a significant behavior among Chinese college students.

Problematic micro-drama viewing reduces academic time and can worsen social withdrawal and emotional distress, negatively impacting college students’ emotional, physical, psychological, and social wellbeing. Elhai et al. (2025) found a link between anxiety, FoMO, and internet addiction. Bhukya and Lakshmana (2025) showed internet addiction worsens health and called for prevention. While online micro-short dramas join education and entertainment, they also create the new problem of “problematic viewing.”

Digital addiction is especially prominent among college students, who are digital natives. Mobile internet growth and free algorithm-driven content foster addiction, with data shaping consumer choices and reducing budget awareness, jeopardizing mental health. Most research focuses on market rise and economy, not on psychological mechanisms of problematic viewing. Exploring these mechanisms has theoretical and practical significance for preventing problematic behaviors and promoting student wellbeing.

Perceived stress, as the cognitive assessment result of an individual’s perception of environmental pressure, is an important distant factor for predicting problematic viewing of micro-short dramas. The General Strain Theory was proposed to explain the deviant behaviors and addictive tendencies of individuals in stressful situations (Agnew, 1992; Agnew and Brezina, 2015). This theory explains how external stressors lead to deviant behaviors through psychological mechanisms, classifying stress into three types: inability to achieve valuable goals, encountering harmful experiences, and losing positive and valuable experiences (Agnew, 1992, 2006). The theory holds that significant stress increases the likelihood of individuals adopting problem behaviors to cope with or avoid these stressors.

Agnew and White (1992) as well as Jang and Johnson (2003) and other researchers have verified the applicability of this theory in explaining deviant behaviors and addictive behaviors through empirical studies. In the field of digital addiction, researchers have analyzed mobile phone addiction (Peng et al., 2022), internet addiction (Jun and Choi, 2015), and short-video addiction (Zhang et al., 2024) using the general stress theory, and have confirmed the significant predictive role of perceived stress in various types of internet addiction behaviors. Specifically, the student population is exposed to multiple stressors such as academic competition, employment pressure, and interpersonal relationship adaptation. According to the general stress model (Agnew, 1992), when individuals fail to meet social needs, are exposed to harmful relationships, or lose interpersonal connections, they will experience negative emotions, which in turn prompts them to engage in problem behaviors such as problematic viewing of micro-short dramas (Ryan and Deci, 2024; Zhang et al., 2024). Moreover, the immediate entertainment function provided by micro-short dramas allows users to choose content to relieve stress. Due to algorithm-driven personalized recommendations, colorful content, short duration, and smooth viewing experience, users in a high-pressure state are more likely to develop addictive tendencies (Huang et al., 2021).

The I-PACE model (Interaction of Person-Affect-Cognition-Execution Model) for problematic internet use behavior indicates that emotional cognitive response factors and individual executive function factors are the key variables leading to internet use problems (Brand et al., 2016). Stress perception, as a subjective perception of the environment, refers to the physical and mental tension and discomfort that individuals experience after receiving threatening stimuli in the environment and conducting cognitive evaluations (Yang and Huang, 2003). Existing studies have confirmed that external environmental stress is a risk factor for individual internet addiction (Zhang and Chen, 2021). When college students perceive stress, they will experience more unease and anxiety, and may then use the internet to relieve stress and negative emotions (Pu et al., 2023), and the use of short-video behavior may become a means to escape from the stress source (Huang et al., 2021). Moreover, research has found that stress perception can predict internet addiction tendencies (Zhang et al., 2016), and the stress perceived by individuals also affects short-video addiction (Liu et al., 2021). When the stress source triggers strong negative emotions and individuals lack healthy coping strategies, people will overuse digital media to alleviate the discomfort caused by stress (Zhang et al., 2024). Broidy (2001) specifically pointed out that college students have a higher tendency to offend and a higher risk of addiction under stress, so they are more likely to adopt ineffective and immature ways to cope with stress sources, such as immersing themselves in the viewing of micro-short dramas to escape real-life stress, thereby forming a dependent pattern.

Boredom (state boredom) plays a crucial intermediary role between the perception of stress and the problematic viewing of micro-short dramas. Boredom is defined as a frustrating tendency characterized by a desire to engage with one’s surroundings or participate in fulfilling activities, which is unfulfilled, a psychological state (Struk et al., 2017). In the field of psychology, it is generally categorized into two types: trait boredom and state boredom (Elpidorou, 2017). State boredom refers to a brief, passive state triggered by specific circumstances, characterized by its immediate onset and specific intensity (Eastwood et al., 2012), while trait boredom is a long-standing personality tendency reflecting an individual’s inherent propensity to experience boredom (Farmer and Sundberg, 1986).

The emergence of boredom stems from three core factors: Firstly, when people generally feel that their lives lack meaning, they will perceive daily activities as both uninteresting and meaningless, thereby reducing their sense of life goals (Elpidorou, 2017); Secondly, if one is unable to interact with the environment or engage in satisfying activities, it will also trigger a state of boredom (Eastwood et al., 2012); Thirdly, attentional dysfunction leads to frequent distraction during daily tasks, thereby increasing the probability of feeling bored (Hsu et al., 2020). For college students in a high-perceived stress state, stressors consume cognitive resources, weakening their ability and motivation to engage in complex activities and increasing their likelihood of falling into state boredom.

According to the Functional Model of Emotion, boredom is a functional adaptive emotion that drives people to seek novel activities and engage in more rewarding pursuits, even if these activities have negative hedonic value (Bench and Lench, 2013, 2019). Bench and Lench (2013) proposed that boredom can be regarded as an emotion with an adaptive function, helping an individual continuously adjust their goal direction, suggesting that giving up the current activity and pursuing more rewarding activities may be more beneficial. From a functional perspective, boredom can be regarded as an exploration signal. When the intensity of the current experience weakens, boredom prompts individuals to seek new alternative experiences to break the psychological monotony. In the context of using micro-short dramas, state boredom as a signal emotion drives individuals to seek immediate stimulation. The strong plot, high-density narrative rhythm, and immediate feedback mechanism of micro-short dramas precisely provide this “emotional compensation,” meeting the needs of bored individuals for novel stimulation.

Research indicates that high levels of boredom in daily life significantly increase adolescents’ addiction to short videos (Lu et al., 2022). Short video addiction is closely related to boredom tendencies among college students (Xie et al., 2023). After studying ordinary users of China’s Douyin app, researchers found that a tendency toward fatigue is closely related to problematic use of Douyin (Yao et al., 2023). Similarly, studies have also linked boredom to internet addiction (Liang et al., 2022; Orsolini et al., 2023; Skues et al., 2016), Facebook addiction (Donati et al., 2022), social media addiction (Allahverdi, 2022), and smartphone addiction (Malaeb et al., 2022; Wang et al., 2020; Wolniewicz et al., 2020). According to the self-control source model, an individual’s self-control is a finite resource that, once depleted, leads to a loss of energy, or self-depletion (Baumeister et al., 2000). Stress management and emotional control consume psychological energy, leading to a shortage of energy in other areas (Tan et al., 2012). When individuals face external stress, they control themselves by consuming psychological resources, thereby reducing their self-control capacity (Xia et al., 2020). Research shows that various stressors can lead to self-exhaustion in individuals (Prem et al., 2016). The finite willpower model proposed by Muraven and Baumeister argues that a reduction in self-control resources leads to uncontrolled behavior in individuals (Muraven and Baumeister, 2000).

Following the occurrence of individual self-depletion, a decline in overall self-control abilities leads to the development of internet addiction behaviors (Ding et al., 2020). Therefore, perceived stress may influence the addiction to micro-short dramas through the mediating role of state boredom: high-pressure environments weaken an individual’s self-control resources and sense of meaning, leading to state boredom; and boredom, as a state of seeking stimulation, encourages individuals to fill their psychological void through the immediate stimuli of micro-short dramas, a cascading pattern has formed, in which higher perceived stress is associated with higher state boredom, which in turn is linked to more problematic viewing of micro-short dramas.

Research based on the Diathesis-Stress Model (Ingram and Luxton, 2005) has shown that feelings of loneliness are not merely a subjective experience of social deprivation, but rather a factor of cognitive vulnerability (chronic cognitive vulnerability). Cacioppo and Hawkley’s (2009) study proposed the Social Monitoring System hypothesis. The research indicates that individuals who experience loneliness are overly vigilant in response to social threats. This persistent state of heightened awareness consumes cognitive resources, ultimately leading to a “cumulative cognitive load effect.

This means that when external pressure intervenes, the cognitive framework of individuals with high levels of loneliness is already in a state of “resource depletion.” According to Self-Determination Theory (Deci and Ryan, 2000), loneliness is essentially a persistent frustration of the need for relatedness, which systematically weakens an individual’s intrinsic motivation and autonomy. Therefore, when facing a stressful situation, individuals with high levels of loneliness not only face the physical depletion of cognitive resources, but also encounter motivational deficits (motivational deficits), making it difficult for them to maintain attentional engagement in the current task, and thus more likely to fall into state boredom described by Eastwood et al. (2012), where they “want to engage but cannot”.

In summary, the loneliness is resolved through cognitive-level resource competition and motivational-level internal drive, and constitutes a “risk amplifier” for the transformation of stress into boredom. This study hypothesizes that the loneliness significantly moderates the relationship between perceived stress and state boredom (first-stage moderation): at high levels of loneliness, the positive predictive effect of stress on state boredom is significantly enhanced; while at low levels of loneliness, this effect weakens or is not significant.

**Figure 1:**
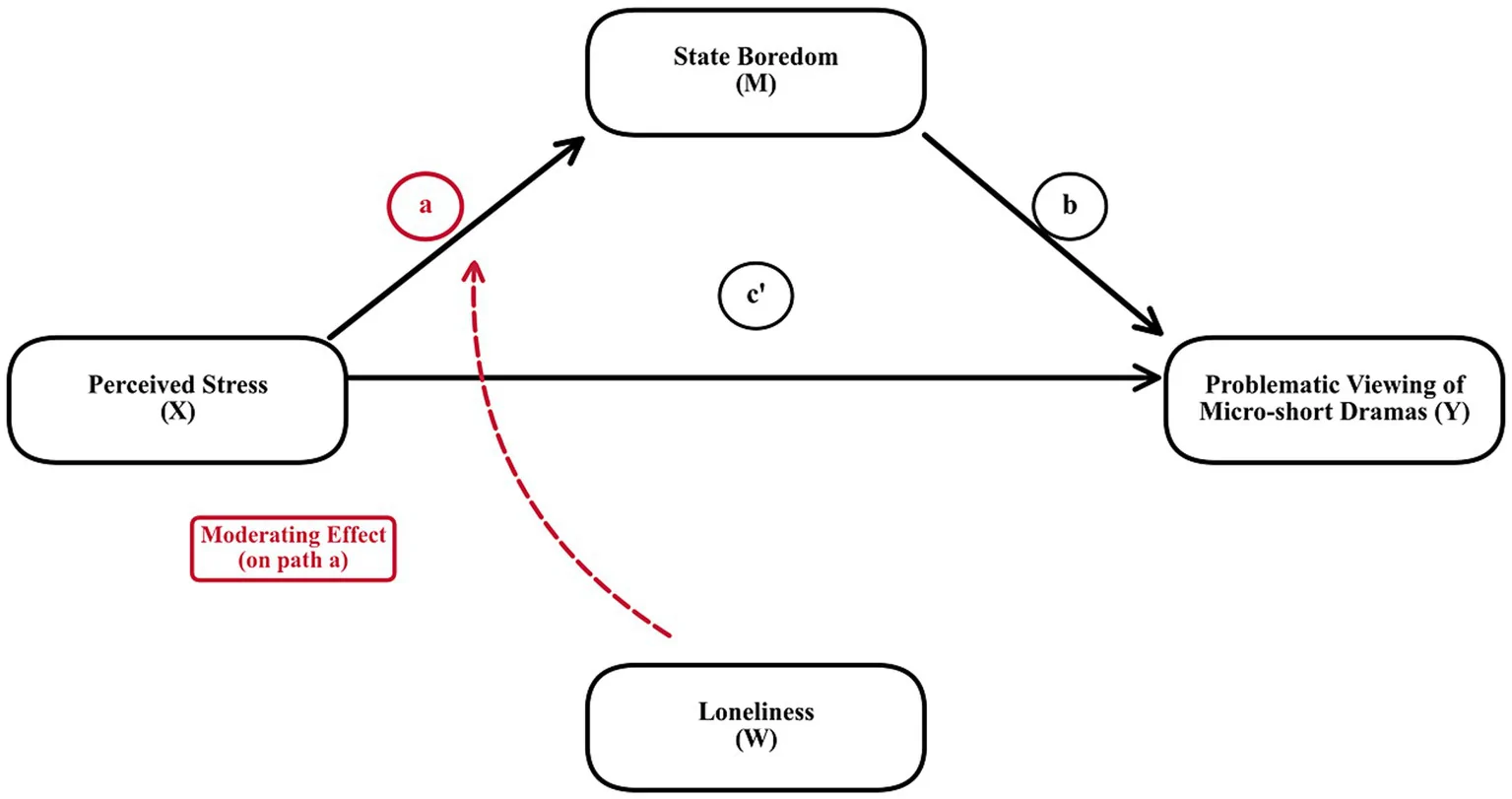
Conceptual moderated mediation model. PROCESS Model 7 (Hayes, 2018). Path a represents the conditional effect of perceived stress on state boredom moderated by loneliness. X, perceived stress, M, state boredom, Y, problematic viewing of micro-short dramas, W, loneliness.

Integrating these perspectives, the present study proposes a hypothesized moderated mediation model (Figure 1). The model combines General Strain Theory (Agnew, 1992), which positions negative emotions as the conduit through which stress is linked to deviant or addictive coping; the Functional Model of Emotion (Bench and Lench, 2013), which specifies boredom as the relevant negative emotional state motivating escape-oriented media use; the I-PACE model (Brand et al., 2016), which locates affective-cognitive responses as the core mechanism linking predisposing and situational factors to problematic internet use; and the vulnerability-stress framework (Ingram and Luxton, 2005), which identifies loneliness as a dispositional vulnerability that conditions the strength of the stress-emotion association. Within this framework, the model specifies how a social factor (loneliness), an emotional state (state boredom), and perceived environmental stress are associated with problematic media use among university students: when high perceived stress and high loneliness coexist, the positive association between perceived stress and state boredom is stronger, which in turn is linked to higher problematic viewing of micro-short dramas. Because the design is cross-sectional, this model represents a hypothesized, theory-derived structure rather than an established causal chain. In sum, the present study examines a hypothesized model in which perceived stress is associated with problematic viewing of micro-short dramas, state boredom statistically mediates this association, and loneliness moderates the first stage of this pathway, mirroring the structure specified in the title of this article. Based on this framework, we propose the following research hypotheses:

*Hypothesis 1*: Perceived stress is significantly positively correlated with problematic viewing of micro-short dramas.

*Hypothesis 2*: State boredom mediates the relationship between perceived stress and problematic viewing of micro-short dramas.

*Hypothesis 3*: Loneliness moderates the relationship between perceived stress and state boredom (first-stage moderation). Specifically, the positive predictive effect of perceived stress on state boredom is significantly stronger among individuals with high loneliness (i.e., high loneliness amplifies the transformational path from stress to boredom).

*Hypothesis 4*: The mediating effect of state boredom is moderated by loneliness, manifested as a moderated mediating effect (moderated mediation). Specifically, the conditional indirect effect of perceived stress on problematic viewing of micro-short dramas via state boredom is significant at high levels of loneliness but not at low levels.

This study makes three contributions. First, micro-short dramas are a rapidly emerging, algorithm-driven media form distinct from both long-form binge-watching and short-video feeds, yet problematic viewing of this form has rarely been studied. Second, whereas prior stress-addiction research has focused on distal traits, the present study examines state boredom as a situationally modifiable mechanism and loneliness as a boundary condition, thereby identifying not only how but also for whom the stress-viewing association is strongest. Third, the findings carry direct implications for stratified, dual-target campus interventions that combine stress management with social connection.

## 2 Materials and methods

### 2.1 Research objectives

This study considers the relationship between perceived stress and problematic viewing of micro-short dramas, testing the mediating role of state boredom and the moderating role of loneliness in this pathway. Specifically, the study considers: Whether state boredom mediates the relationship between perceived stress and problematic viewing of micro-short dramas; Whether loneliness moderates the first stage of the stress-boredom pathway, thereby forming a moderated mediation model (PROCESS macro model 7).

### 2.2 Participants

Participants were recruited through convenience sampling from five domestic universities. All participants provided informed consent, were informed of the survey’s anonymity and the academic-only use of the data, and received compensation upon completing valid questionnaires. Inclusion criteria were: (1) Full-time undergraduate students; (2) Experience watching micro-short dramas within the past 3 months (a screening question on the questionnaire cover page—“Have you watched online micro-short dramas?”—automatically redirected those selecting “Never watched” to the exit page and excluded them). A total of 821 questionnaires were distributed, yielding 781 valid responses, representing a valid response rate of 95.1%. The average age of participants was 21.1 ± 1.6 years; 384 participants (49.2%) were male, and 397 (50.8%) were female.

### 2.3 Research instruments

#### 2.3.1 Micro-short drama problematic viewing questionnaire

This study employs the Binge-Watching Addiction Questionnaire (BWAQ), developed by Forte et al. (2021), as the foundational framework and adapts it to the characteristics of micro-short dramas. Given the fundamental differences between micro-short dramas and long-form dramas in terms of narrative pacing (high frequency of exciting moments), content distribution (algorithm-driven recommendations), and the original scale’s inability to fully capture these mechanisms, the original questionnaire was renamed the Addiction Questionnaire. However, considering the continuum of behavioral addictions (Kardefelt-Winther, 2014) and to avoid the overly pathological connotations of the term “addiction” in the Chinese context, the study uses the term “problematic viewing” to refer to this construct. This term emphasizes its role as a transitional state between normal entertainment and maladaptive behavior. The research team retained the original BWAQ’s four dimensions (craving, dependence, expectation, avoidance) and added five items related to “media characteristics” (two items on algorithm dependence and three items on the dependence on exciting moments), resulting in a 25-item Chinese version of the micro-drama problematic viewing questionnaire (see Appendix A). The scale uses a 5-point frequency rating (0 = never, 4 = always), with a total score ranging from 0 to 100. Higher scores indicate a greater degree of problematic viewing.

The revision and validation of the scale was divided into two stages: the first stage recruited 172 subjects for exploratory factor analysis (EFA); The second phase applied an optimized scale of EFA to 781 formal samples for validation factor analysis (CFA) and subsequent hypothesis testing: (1) Content effectiveness: invited four experts (film and television communications, Psychological measurements, applied language domain) evaluated subject-idea matching on a four-level scale, showing S-CVI/Ave = 0.98, I-CVI in the range of 0.75–1.00 (*M* = 98), and Kappa coefficient averaging 0.97, indicating good content effectiveness. (2) Structural effectiveness: EFA was first performed on 172 subjects, KMO = 0.87, the Bartlett spherical test was significant (*p* < 0.001), 5 factors were extracted to accumulate 76.73% of the variation, and the factor load of each entry was 0.71–0.88; Then the formal sample (*n* = 781) was validated by CFA, and the five-factor model fit well (*χ*^2^/*df* = 2.96, RMSEA = 0.05[90%CI:0.046–0.054], CFI = 0.91, TLI = 0.90, SRMR = 0.04). It was significantly better than single-factor model (Δ*χ*^2^ = 355.39, *p* < 0.001) and four-factor model (Δ*χ*^2^ = 262.07, *p* < 0.001), confirming the independence of the dimensions of media characteristics.

The reliability analysis indicates that the total scale has a Cronbach’s *α* of 0.91, with *α* coefficients ranging from 0.63 to 0.79 for each dimension. The composite reliability (CR) is all greater than 0.75, and the average variance extracted (AVE) ranges from 0.34 to 0.73. It is worth noting that the AVE value for the desire dimension is 0.34, which is slightly lower than the traditional threshold of 0.50 suggested by Fornell and Larcker (1981). This may be due to the complex cognitive-emotional processes measured by this dimension, such as psychological craving and dependence. However, the CR value for this dimension is greater than 0.75 and meets the internal consistency criterion proposed by Bagozzi and Yi (1988). According to the recommendations for exploratory research by Hair et al. (2019), when CR is sufficiently high and item loadings are acceptable, a slight deficiency in AVE does not constitute a fundamental measurement defect. The formal sample (*n* = 781) exceeds common CFA sample-size requirements (subject-to-item ratio of approximately 31:1; Kline, 2016), providing adequate statistical power for the validity analyzes; a sensitivity analysis (alpha = 0.05, *n* = 781) further indicated that the moderated mediation model could detect interaction effects as small as *f*^2^ = 0.01 with 80% power, well below the observed interaction effect (*f*^2^ = 0.04, post-hoc power >0.99). Taken together, the content-validity indices, the EFA and CFA results from two independent samples, and the internal-consistency and composite-reliability estimates indicate that the adapted questionnaire is a psychometrically sound instrument for assessing problematic viewing of micro-short dramas among Chinese university students.

#### 2.3.2 UCLA loneliness scale

This study used the UCLA Loneliness Scale-Version 3 (ULS-20) developed by Russell (1996). The Chinese version was translated and revised by Liu (1999). The scale comprises 20 items, 10 of which are reverse-scored, and features a unidimensional design. The scale uses a 4-point frequency scale (1 = Never, 2 = Rarely, 3 = Sometimes, 4 = Always), with higher total scores indicating greater loneliness. Representative items include “Do you often feel that your relationships with others are harmonious?” (reverse-scored) and “Do you often feel a lack of fellowship?” The Cronbach’s *α* coefficient for this scale in the present study was 0.95.

#### 2.3.3 Multidimensional state boredom scale

This study employed the Multidimensional State Boredom Scale (MSBS), developed by Fahlman et al. (2013), which was translated and revised into Chinese by Liu et al. (2013). The scale is based on five dimensions: lack of attention, time perception, low arousal, high arousal, and detachment, with a total of 24 items. The scale uses a 7-point Likert scale (1 = strongly disagree, 7 = strongly agree), with higher total scores indicating a more boring current state. Typical questions include “Time seems to pass more slowly than usual” (time perception dimension) and “It’s difficult for me to focus my attention” (lack of attention dimension). In this study, the Cronbach’s *α* coefficient for the MSBS was 0.96.

#### 2.3.4 Perceived stress scale

This study employed the Perceived Stress Scale (PSS-10) developed by Cohen et al. (1983). The Chinese version, known as the Chinese Perceived Stress Scale (SCPSS-10), was translated and revised by Lu et al. (2017). The scale consists of 10 items, which were extracted through exploratory factor analysis to yield a two-factor structure: negative feelings (6 items) and positive feelings (4 items). The scale uses a 5-point scoring system (0 = not at all, 4 = always), with items 4, 5, 7, and 8 being reverse-scored. Higher total scores indicate a higher level of perceived stress. Typical questions include “How much time have you felt upset over unexpected events over the past month?” and “How much time have you felt unable to control important aspects of your life over the past month?” In this study, the Cronbach’s *α* coefficient for the scale was 0.91.

### 2.4 Ethics statement

The study followed the Declaration of Helsinki. Formal ethical review was waived by the institutional review boards of Chengdu Jincheng College, Chengdu College of Arts and Sciences, and Beijing Normal University due to the anonymous survey design. All participants provided written informed consent.

### 2.5 Statistical analysis

Data analysis was performed using SPSS 27.0 and the PROCESS macro. First, Harman’s single-factor test was used to assess common method bias, supplemented by a CFA-based unmeasured latent method factor model (Podsakoff et al., 2012). Subsequently, the distributional properties (skewness, kurtosis, and Shapiro–Wilk and Kolmogorov–Smirnov tests) were examined for all variables (perceived stress, state boredom, loneliness, and problematic micro-short drama viewing). Appropriate statistical techniques were selected based on data distribution features to analyze demographic variables and between-group differences in study variables. Furthermore, suitable correlation coefficients were identified and applied to evaluate bivariate relationships among all study variables. Finally, Hayes’ PROCESS macro version 4.2 was used to test the hypothesized moderated mediation model.

In this study, Hayes’ PROCESS Model 7 was used to simultaneously test the mediating role of state boredom in the association between perceived stress and problematic micro-short drama viewing and whether this indirect association is conditioned by loneliness. This model allows for reporting conditional indirect effects within a single unit of analysis (Hayes, 2018). Specifically, the model tested whether loneliness moderated the initial path from perceived stress to state boredom, that is, whether the association between stress and boredom was stronger at higher levels of loneliness. Conditional indirect and direct effects were estimated using 5,000 bootstrap iterations to obtain 95% confidence intervals (CIs). An effect was considered statistically significant if its 95% CI (defined by the lower confidence limit, LLCI, and upper confidence limit, ULCI) did not include zero. All continuous variables were mean-centered prior to analysis.

## 3 Results

In addition, common method bias was examined with a CFA-based unmeasured latent method factor model in which all 79 items loaded on both their theoretical trait factor and a common method factor. A single-factor model fit the data very poorly (*χ*^2^ = 15,088.75, *df* = 3,002, CFI = 0.625, TLI = 0.615, RMSEA = 0.072), whereas the hypothesized four-factor model fit well (*χ*^2^ = 4,711.30, *df* = 2,996, CFI = 0.947, TLI = 0.945, RMSEA = 0.027). Adding the latent method factor produced only a marginal improvement in fit (*χ*^2^ = 4,196.17, *df* = 2,917, CFI = 0.960, RMSEA = 0.024; ΔCFI = 0.014, ΔRMSEA = 0.003). Critically, the method factor accounted on average for only 1.6% of item variance (mean of squared standardized method loadings), compared with 44.3% accounted for by the substantive trait factors, and all trait loadings remained statistically significant and virtually unchanged after controlling for the method factor. These results indicate that common method bias is unlikely to account for the observed associations (Podsakoff et al., 2012).

All participants were university students with experience of watching micro-short dramas within the past 3 months. The sample consisted of 384 males (49.17%) and 397 females (50.83%), with an average age of 21.10 ± 1.56 years. Due to the self-report method used to collect data in this study, there is a potential for common method bias. A Harman single-factor test was conducted to examine this, and the results showed a KMO value of 0.898, a Bartlett’s spherical test significance *p* < 0.001, and five factors with initial eigenvalues greater than 1. The first common factor explained 28.943% of the variance, which is below the 40% threshold (Podsakoff et al., 2012). This result is consistent with an absence of severe common method bias; given the limited sensitivity of Harman’s test, this was further examined with a CFA-based approach (see below).

A multivariate collinearity diagnostic was performed with problematic viewing micro-short dramas as the dependent variable. Variance inflation factors (VIF) were 1.08 for perceived stress, 2.16 for state boredom, and 2.09 for loneliness. All values were below 5 (and below 10), indicating no significant multicollinearity issues. Normality was assessed using Kolmogorov–Smirnov tests, complemented by skewness and kurtosis statistics. Results indicated that problematic viewing micro-short dramas (*z* = 0.056, *p* = 0.014) and loneliness (*z* = 0.067, *p* = 0.002) significantly deviated from normal distribution. Absolute skewness (0.05–0.58) and kurtosis (−0.35 to 0.02) values were all well below conventional thresholds (|skew| <2, |kurtosis| <7), indicating no severe departures from normality; Shapiro–Wilk tests were significant for all variables, and Kolmogorov–Smirnov tests were significant for problematic viewing and loneliness, a common outcome with large samples. We therefore adopted a conservative analytic strategy: non-parametric tests for group comparisons, Spearman’s rank correlations for bivariate associations, and bias-corrected bootstrap confidence intervals (5,000 resamples) for all indirect and conditional effects, as bootstrapping makes no distributional assumptions (Hayes, 2018). Therefore, Mann–Whitney U tests were used to analyze significant differences between the two categorical variables (gender; *Z* values are shown in Table 1). Kruskal–Wallis H tests were employed for the multinomial variables (major field of study, grade level). The descriptive statistics for the demographic variables and the results of the differences tests for problematic viewing, perceived stress, state of boredom, and feelings of loneliness are presented in Table 1.

**Table 1:** Demographic characteristics and group differences in problematic viewing of micro-short dramas, loneliness, state boredom, and perceived stress (*N* = 781).

Variables	*n*	%	Problematic viewing of micro-short dramas	Loneliness	State boredom	Perceived stress
Gender						
Male	384	49.17	47.91 ± 15.78	38.23 ± 10.97	102.59 ± 29.97	29.74 ± 7.41
Female	397	50.83	48.20 ± 15.72	36.94 ± 10.92	102.22 ± 29.91	29.98 ± 7.74
*Z* value			−0.428	1.670	−0.086	−0.608
Academic track						
Liberal arts	368	47.12	48.37 ± 16.30	37.72 ± 11.50	101.56 ± 31.33	29.58 ± 7.62
Science	413	52.88	47.78 ± 15.24	37.44 ± 10.45	103.15 ± 28.63	30.12 ± 7.53
*H* value			0.44	0.01	0.97	1.07
Grade						
Freshman	26	3.33	45.42 ± 14.71	37.58 ± 11.79	103.12 ± 31.54	28.54 ± 7.78
Sophomore	320	40.97	47.33 ± 15.77	37.59 ± 10.79	100.41 ± 29.78	29.09 ± 7.39
Junior	131	16.77	47.86 ± 15.72	39.08 ± 10.67	105.29 ± 28.83	29.22 ± 6.99
Senior	304	38.92	49.14 ± 15.81	36.90 ± 11.16	103.19 ± 30.40	31.07 ± 7.86
*H* value			2.74	5.18	3.00	10.98^*^

^***^p < 0.001; ^**^p < 0.01; ^*^p < 0.05. *n*, sample size; *Z* value, *Z* test value converted from Mann–Whitney *U* test value; *H* value, Kruskal–Wallis test value. Gender: 1 = male, 2 = female; Academic track: 1 = liberal arts, 2 = science; Grade: 1 = freshman, 2 = sophomore, 3 = junior, 4 = senior.

As shown in Table 1, there were no significant differences in problematic viewing of micro-short dramas (*U* = 74,876.00, *p* = 0.669), feelings of loneliness (*U* = 81,486.50, *p* = 0.095), state of boredom (*U* = 75,952.00, *p* = 0.931), and perceived stress (*U* = 74,307.00, *p* = 0.543) based on gender. There were also no significant differences based on professional category in problematic viewing (*H* = 0.44, *p* = 0.508), feelings of loneliness (*H* = 0.01, *p* = 0.922), state of boredom (*H* = 0.97, *p* = 0.324), and perceived stress (*H* = 1.07, *p* = 0.300). However, there was a significant difference in perceived stress based on grade level (*H* (3, *N* = 781) = 10.98, *p* = 0.012); Dunn-Bonferroni post-hoc comparisons indicated that seniors reported higher perceived stress than sophomores (adjusted *p* = 0.015; mean ranks: senior = 424.2 vs. sophomore = 369.5), whereas the remaining pairwise contrasts were not significant (freshman-senior adjusted *p* = 0.776; junior–senior adjusted *p* = 0.193); the effect size was small (epsilon-squared = 0.01). There were no significant differences in problematic viewing (*H* = 2.74, *p* = 0.433), loneliness (*H* = 5.18, *p* = 0.159), or state of boredom (*H* = 3.00, *p* = 0 0.391) based on grade level.

Given that the data did not follow a normal distribution, Spearman’s rank correlation analysis was used to assess associations among the measured variables. Table 2 presents the Spearman correlation matrix for all measured variables. The correlation analysis showed that perceived stress showed a significant positive correlation with problematic micro-short drama viewing (*rs* = 0.352, *p* < 0.01) and a significant positive correlation with state boredom (*rs* = 0.150, *p* < 0.01), but no significant correlation with loneliness (*rs* = −0.004, *p* = 0.918); State boredom showed a significant positive correlation with problematic micro-short drama viewing (*rs* = 0.378, *p* < 0.01) and with loneliness (*rs* = 0.712, *p* < 0.01); loneliness also showed a significant positive correlation with problematic micro-short drama viewing (*rs* = 0.292, *p* < 0.01). Although perceived stress and loneliness showed no significant correlation in this study, this does not preclude loneliness from functioning as a moderator variable (Baron and Kenny, 1986; Fang et al., 2022).

**Table 2:** Spearman correlations for problematic viewing of micro-short dramas, perceived stress, state boredom, and loneliness (*N* = 781).

Variable	1	2	3	4
1. Problematic viewing of micro-short dramas	1			
2. Perceived stress	0.352^**^	1		
3. State boredom	0.378^**^	0.150^**^	1	
4. Loneliness	0.292^**^	−0.004	0.712^**^	1

^***^p < 0.001; ^**^p < 0.01; ^*^p < 0.05. Values are Spearman’s rank correlation coefficients (*rs*).

### 3.1 Moderated mediation model testing (single PROCESS Model 7 framework)

Based on test results comparing demographic variables with the four scales, gender, age, grade level, major, and place of origin were included as control variables. As the first step of the Model 7 analysis, the constituent (unmoderated) pathways were estimated: the total and direct associations of perceived stress with problematic viewing of micro-short dramas, the association of perceived stress with state boredom, and the association of state boredom with problematic viewing. All study variables were mean-centered prior to analysis. Results are presented in Tables 3, 4.

**Table 3:** Regression results for the hypothesized moderated mediation model (PROCESS Model 7).

Outcome variable	Predictor variables	*R* ^2^	*F*	*β*	SE	*t*
Problematic viewing of micro-short dramas	Constant	0.194	31.052^***^	21.146^***^	2.084	10.145^***^
	Perceived stress			**0.433** ^***^	**0.033**	**13.312** ^***^
	Gender			0.001	0.032	0.027
	Age			0.094	0.053	1.766
	Grade			−0.067	0.053	−1.265
	Major			−0.035	0.032	−1.078
	Background			0.015	0.032	0.455
Problematic viewing of micro-short dramas	Constant	0.312	49.997^***^	6.298^**^	2.32	2.714^**^
	Perceived stress			**0.369** ^***^	**0.031**	**12.060** ^***^
	State boredom			**0.349** ^***^	**0.030**	**11.494** ^***^
	Gender			0.004	0.030	0.144
	Age			0.075	0.049	1.525
	Grade			−0.058	0.049	−1.173
	Major			−0.042	0.030	−1.393
	Background			0.010	0.030	0.350
State boredom	Constant	0.037	4.940^***^	80.746^***^	4.331	18.645^***^
	Perceived stress			**0.183** ^***^	**0.036**	**5.155** ^***^
	Gender			−0.010	0.035	−0.278
	Age			0.054	0.058	0.928
	Grade			−0.027	0.058	−0.471
	Major			0.019	0.035	0.551
	Background			0.012	0.035	0.344

^***^p < 0.001; ^**^p < 0.01; ^*^p < 0.05. *R*^2^, coefficient of determination; *F*, *F* statistic; *β*, standardized regression coefficient; SE, standard error; *t*, *t* statistic. For constant rows, values in the *β* column are unstandardized intercepts. Bold values indicate statistical significance.

**Table 4:** Direct, indirect, and total associations in the mediation component of the model (bootstrap = 5,000).

Effect	*B*	95% CI	SE	*p*	Percentage (%)
Total effect, *c*	0.905	(0.759, 1.043)	0.067	<0.001	
Direct effect, *c*′	0.769	(0.636, 0.895)	0.063	<0.001	84.95
Indirect effect, *a* × *b*	0.136	(0.080, 0.199)	0.031	<0.001	15.03
*a* path (*X* → *M*)	0.739	(0.455, 1.032)	0.139	<0.001	
*b* path (*M* → *Y*)	0.184	(0.152, 0.215)	0.016	<0.001	

Bootstrap confidence intervals based on 5,000 resamples. X, perceived stress; M, state boredom; Y, micro-short drama problematic viewing. Percentage indicates the proportion of total effect.

Table 3 indicates that, after controlling for demographic variables, perceived stress significantly and positively predicted problematic micro-short drama viewing (*β* = 0.433, *t* = 13.312, *p* < 0.001) and state boredom (*β* = 0.183, *t* = 5.155, *p* < 0.001); State boredom significantly and positively predicted problematic micro-short drama viewing (*β* = 0.349, *t* = 11.494, *p* < 0.001). According to Table 3, the R^2^ for the mediation model including the mediating variable was 0.312 (*F* = 49.997, *p* < 0.001), while the R^2^ for the total effects model was 0.194 (*F* = 31.052, *p* < 0.001).

As shown in Table 4, the mediation effect analysis showed that the total effect of perceived stress on problematic viewing of micro-short dramas was significant, *B* = 0.905, 95% CI [0.759, 1.043]. After incorporating boredom as a mediating variable, the direct effect remained significant (*B* = 0.769, 95% CI [0.636, 0.895]). The indirect effect was tested via 5,000 Bootstrap samples, yielding *B* = 0.136 (95% CI: [0.080, 0.199]). Since the confidence interval did not include zero, the mediating effect was significant, accounting for 15.03% of the total effect. The proportion of the indirect association (15.03%) was computed from the unstandardized effects (0.136/0.905), as is conventional in PROCESS reporting.

### 3.2 Moderating effects of loneliness

Under the conditions of controlling for gender, age, grade level, major, and geographical origin, the PROCESS macro model 7 was employed, with problematic viewing of micro-short dramas serving as the dependent variable, perceived stress as the independent variable, state boredom as the mediating variable, and loneliness as the moderating variable (specifically, the path through which perceived stress influences state boredom). The results are presented in Table 5, which shows that the interaction between perceived stress and loneliness significantly predicts state boredom (*β* = 0.138, *t* = 5.643, *p* < 0.001), indicating that loneliness moderates the relationship between perceived stress and state boredom.

**Table 5:** Moderation model: regression of state boredom on perceived stress, loneliness, and their interaction (PROCESS Model 7, mediator equation).

Outcome variable	Predictor variables	*R* ^2^	*F*	*β*	SE	*t*
State boredom	Constant	0.541	129.984^***^	102.401^***^	1.071	95.607^***^
	Perceived stress			0.183^***^	0.025	7.423^***^
	Loneliness			0.711^***^	0.025	29.326^***^
	Gender			−0.022	0.036	−0.612
	Age			0.036	0.059	0.608
	Grade			−0.042	0.059	−0.712
	Major			0.029	0.036	0.806
	Background			0.018	0.036	0.500
State boredom	Constant	0.559	122.255^***^	102.401^***^	1.046	97.897^***^
	Perceived stress			0.189^***^	0.036	7.831^***^
	Loneliness			0.704^***^	0.025	29.326^***^
	Perceived stress × loneliness			0.138^***^	0.024	5.643^***^
	Gender			−0.022	0.036	−0.612
	Age			0.036	0.059	0.608
	Grade			−0.042	0.059	−0.712
	Major			0.029	0.036	0.806
	Background			0.018	0.036	0.500

^***^p < 0.001; ^**^p < 0.01; ^*^p < 0.05. *R*^2^, coefficient of determination; *F*, *F* statistic; *β*, standardized regression coefficient; SE, standard error; *t*, *t* statistic. For constant rows, values in the *β* column are unstandardized intercepts.

**Figure 2:**
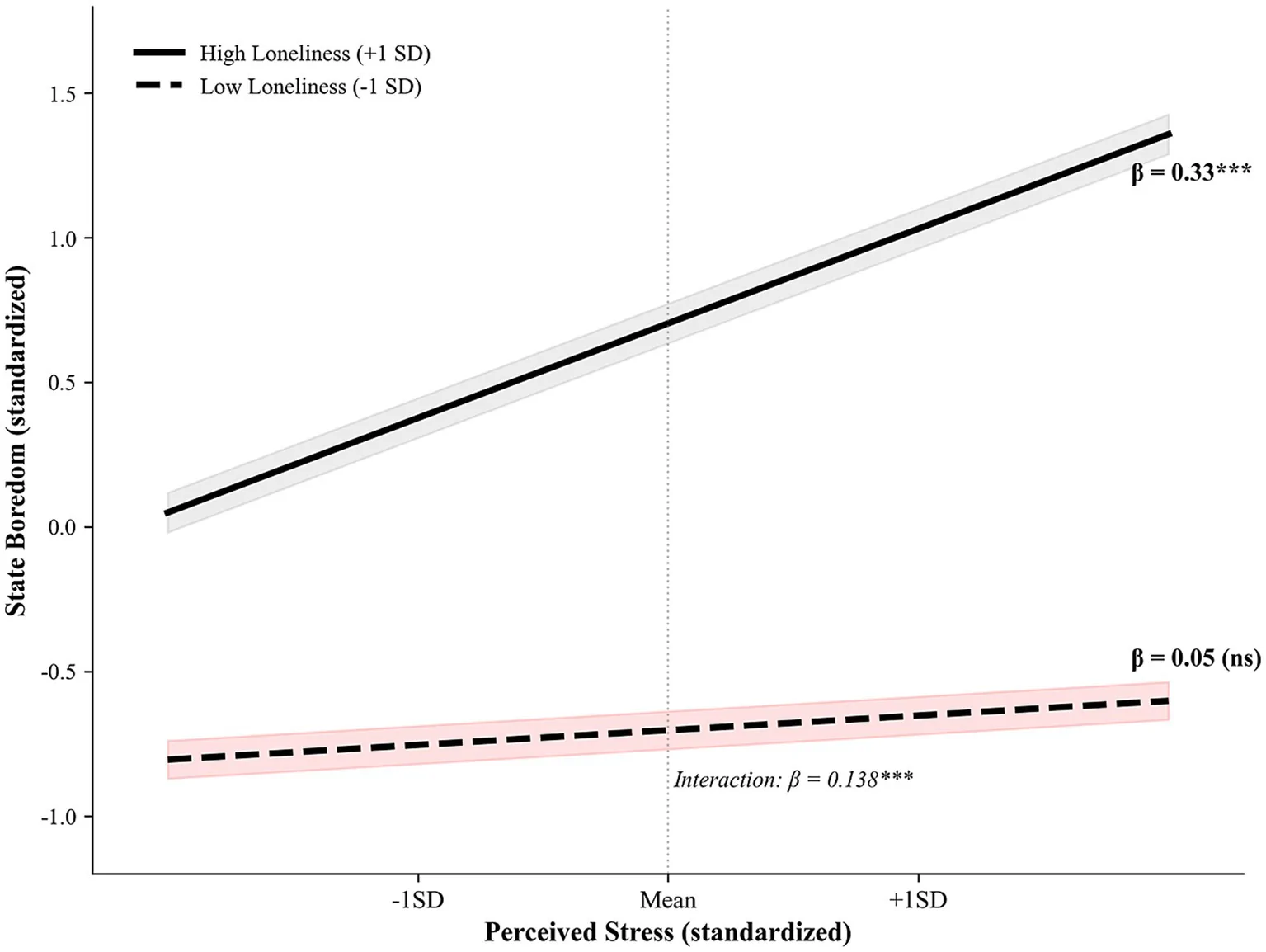
The moderating effect of loneliness on perceived stress and state boredom. ^***^p < 0.001, (ns) *p* > 0.05. Error bands represent 95% confidence intervals.

To clarify this moderation effect, the association between perceived stress and state boredom was plotted separately for low (*M* − 1 SD) and high (*M* + 1 SD) levels of loneliness (see Figure 2). Simple slope analysis showed that perceived stress significantly predicted state boredom at high levels of loneliness (*β* = 0.327, *p* < 0.001) but not at low levels of loneliness (*β* = 0.051, *p* = 0.130). The results show that loneliness increases the effect of perceived stress on state boredom, specifically appearing as an important stress-boredom association only among high-loneliness individuals, whereas no significant association exists among low-loneliness individuals.

**Figure 3:**
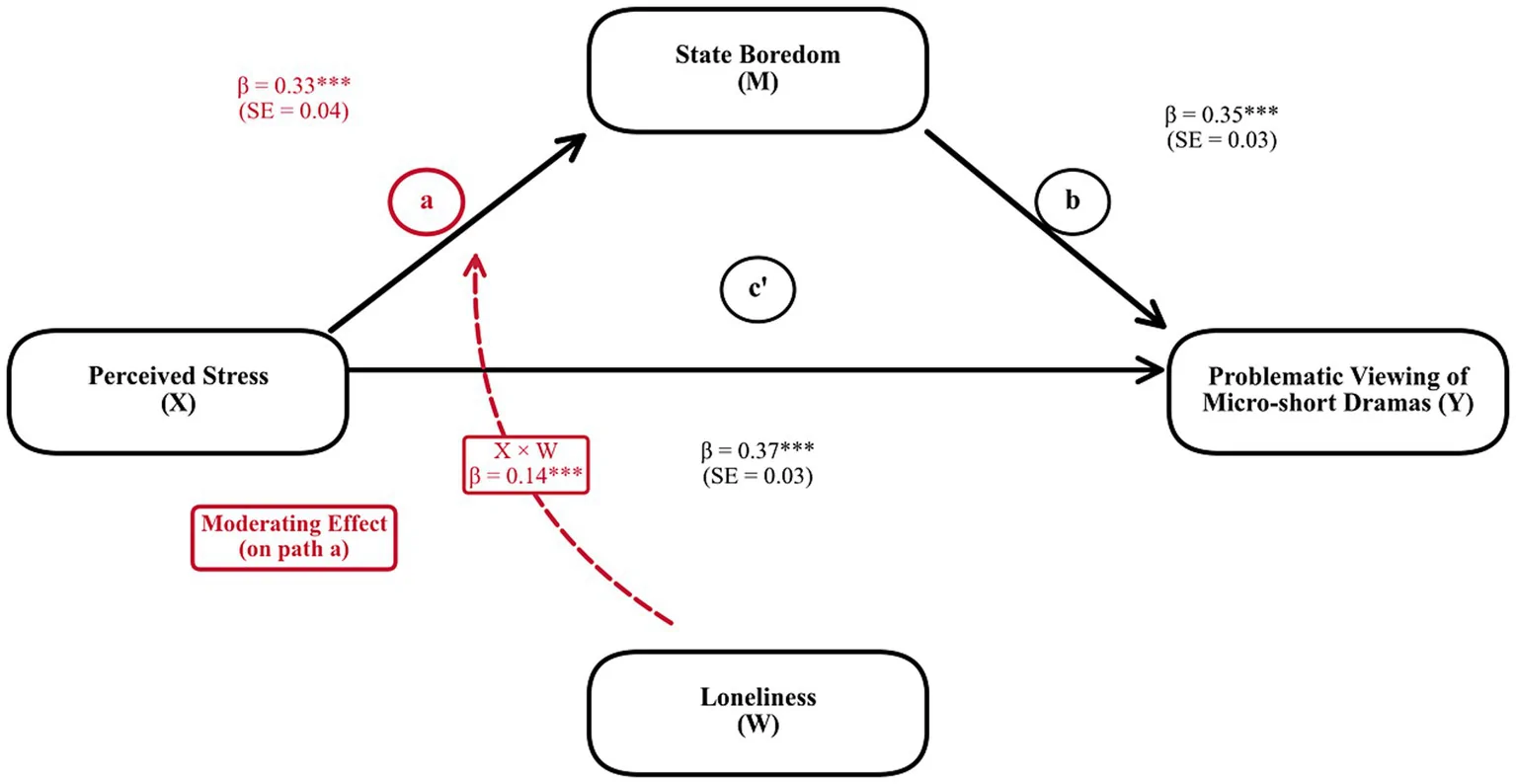
Path analysis results of the moderated mediation model. Standardized coefficients are displayed. The path coefficients in this figure have been redrawn to match the values reported in the tables (*a* = 0.183, *b* = 0.349, direct effect = 0.369). PROCESS Model 7 (Hayes, 2018). *N* = 781. All coefficients are standardized (*β*) and correspond exactly to the values reported in the tables. The dashed arrow indicates the moderating effect of loneliness on path a (interaction *X* × *W*). X, perceived stress, M, state boredom, W, loneliness, Y, problematic viewing of micro-short dramas. ^*^p < 0.05; ^**^p < 0.01; ^***^p < 0.001.

Additionally, the conditional indirect effects and the index of moderated mediation were examined to test Hypothesis 4. Results (see Figure 3) revealed that the conditional indirect effect of perceived stress on problematic micro-short drama viewing via state boredom was significant at high loneliness levels (effect = 0.114, 95% CI [0.085, 0.145]) but not at low loneliness levels (effect = 0.017, 95% CI [−0.006, 0.043]). Critically, the index of moderated mediation was statistically significant (standardized index = 0.048, 95% bootstrap CI [0.030, 0.068]), confirming that the indirect association differed significantly across levels of loneliness. Specifically, the indirect association of perceived stress with problematic micro-short drama viewing via state boredom was significant only among individuals with high loneliness and increased with higher levels of loneliness (from 0.017 to 0.114).

## 4 Discussion

### 4.1 Relationship between perceived stress and problematic viewing of micro-short dramas

This study identified that perceived stress is a significant positive predictor of problematic viewing of micro-short dramas among Chinese college students, supporting Hypothesis 1. This finding is consistent with the core proposition of the General Strain Theory (GST), which posits that when individuals encounter adverse circumstances such as unattainable important goals, harmful stimuli, or loss of positive experiences, and lack legitimate coping resources, they are more likely to adopt problem behaviors or addictive behaviors as coping strategies (Agnew, 1992, 2006). In the digital media context, micro-short dramas, through their algorithm-driven, precise recommendations and high-density narrative “cliffhangers,” provide an immediate, accessible channel for emotional relief for individuals in a high-pressure state (Huang et al., 2021).

This study extends previous research on general internet addiction (Jun and Choi, 2015) and short-video addiction (Liu et al., 2021) to a specific media form, namely micro-short dramas, and reveals the core role of stress avoidance motivation in driving the consumption of immersive content on vertical screens. According to the I-PACE model (Brand et al., 2016), perceived stress, as a distant vulnerability factor, increases an individual’s susceptibility to specific internet usage disorders through disordered emotional-cognitive responses. It is notable that the effect sizes observed in this research model indicate that direct and indirect associations, suggesting the need to further explore the psychological mechanism by which distant stress is transformed into proximal problematic behaviors.

### 4.2 Mediating role of state boredom

The study findings indicate that state boredom plays a significant mediating role between perceived stress and problematic viewing of micro-short dramas, thereby supporting Hypothesis 2. The pattern in which perceived stress is associated with problematic micro-drama viewing through state boredom can be explained through the Functional Model of Emotion: boredom, as an emotion with adaptive functions, prompts individuals to actively seek novel stimuli to break their mental state (Bench and Lench, 2013, 2019). However, under high levels of perceived stress, an individual’s self-regulation resources are depleted as a response to the stress (ego depletion; Baumeister et al., 2000; Muraven and Baumeister, 2000), leading to a state of “passive boredom” characterized by a desire to engage in fulfilling activities but an inability to do so due to insufficient cognitive resources (Eastwood et al., 2012). micro-short dramas effectively provide “emotional compensation” through their low cognitive load and high immediate feedback, thereby alleviating this state of boredom.

This study posits that state boredom (rather than trait boredom) serves as a critical mechanism. As Tam et al. (2021) have noted, state boredom reflects situational failures in attention and a lack of meaning. Additionally, Bielecki and Kaczmarek (2022) found that the interaction between boredom and insufficient self-control significantly predicts problematic smartphone use.

### 4.3 Moderating role of loneliness

The moderated mediation analysis indicates that loneliness significantly moderates the association between perceived stress and state boredom, such that the association is stronger at higher levels of loneliness (the first half of the moderation). This supports Hypotheses 3 and 4. Specifically, the positive correlation between stress and boredom was significant at high levels of loneliness (*β* = 0.327, *p* < 0.001), but not at low levels (*β* = 0.051, *p* = 0.130). This pattern suggests that loneliness is not a direct risk factor in this model but acts as a contextual amplifier, strengthening the connection between stress and boredom.

Further analysis of the effect revealed that perceived stress is associated with problematic viewing partly via its association with state boredom, with this indirect pathway being significant only among individuals with higher levels of loneliness (effect value = 0.114, 95% CI [0.085, 0.145]). This effect was not significant among individuals with lower levels of loneliness (effect value = 0.017, 95% CI [−0.006, 0.043]). This suggests that for individuals with extensive social interactions, stress is less likely to co-occur with boredom and, correspondingly, shows little association with problematic viewing of micro-short dramas; however, for lonely individuals, stress is more likely to co-occur with situational boredom, which is in turn linked to escapist viewing behaviors.

Notably, perceived stress and loneliness were essentially uncorrelated in this sample (*rs* = −0.004, *p* = 0.918), although each was associated with state boredom and problematic viewing. Several points deserve emphasis. First, a moderator is not required to correlate with the predictor or the outcome; moderation concerns whether the strength of an association varies across levels of the moderator, and near-zero predictor-moderator correlations are in fact statistically desirable because they minimize collinearity between the interaction term and its components (Baron and Kenny, 1986). Second, the near-zero bivariate correlation is theoretically plausible in the present framework: within the vulnerability-stress perspective (Ingram and Luxton, 2005), loneliness is conceptualized as a relatively stable dispositional vulnerability (a diathesis) rather than as a consequence of current situational stress, so diathesis and stressor need not covary in a non-clinical student sample. Third, this independence strengthens the interpretation of the interaction: loneliness does not simply proxy for higher stress exposure but identifies individuals whose stress-boredom association is conditionally stronger. Finally, we acknowledge that the null correlation may partly reflect restriction of range and the situational specificity of the PSS-10 (past-month appraisal) relative to the more trait-like UCLA loneliness measure, and we note this as a question for future longitudinal research.

Theoretically, this finding supports the Vulnerability-Pressure Model (Ingram and Luxton, 2005): Loneliness, as a chronic cognitive vulnerability, consumes regulatory resources through excessive vigilance to social threats (Cacioppo and Hawkley, 2009), leaving individuals with insufficient resources to cope with additional stressors. Therefore, lonely individuals experience a stronger state of boredom under stress, driving them to seek immediate emotional compensation through micro-short dramas. It is noteworthy that loneliness only moderates the first half of the path (stress → boredom), but not the second half (boredom → viewing), suggesting that is associated with whether stress co-occurs with boredom, but once boredom occurs, its transformation process is not affected by loneliness.

State boredom and loneliness are conceptually distinct constructs. Loneliness is a social-evaluative experience arising from perceived deficits in the quality or quantity of one’s social relationships (Russell, 1996; Cacioppo and Hawkley, 2009), whereas state boredom is an attentional-motivational state defined by the inability to engage meaningfully with the current activity or environment despite the desire to do so (Eastwood et al., 2012; Fahlman et al., 2013). One can feel lonely without being bored (e.g., while engaged in meaningful solitary work) and bored without being lonely (e.g., during a monotonous group activity). Their correlation is expected because chronically unmet social needs reduce access to stimulating activities, and both states share negative affect as a common core.

We examined the discriminant validity of state boredom and loneliness in three ways. First, a two-factor CFA (MSBS items and ULS-20 items loading on separate factors) fit the data well (*χ*^2^ = 1,449.09, *df* = 901, CFI = 0.975, TLI = 0.974, RMSEA = 0.028) and significantly better than a one-factor model in which all items loaded on a single factor (*χ*^2^ = 4,131.07, *df* = 902, CFI = 0.853, RMSEA = 0.068; Δ*χ*^2^(1) = 2,681.99, *p* < 0.001). Second, the latent correlation between the two factors was 0.744, below the 0.85 criterion for discriminant validity (Kline, 2016). Third, the heterotrait-monotrait ratio of correlations (HTMT) was 0.736, below the conservative 0.85 threshold (Henseler et al., 2015); HTMT has been shown to outperform traditional criteria such as the Fornell-Larcker criterion, which exhibits low detection rates when indicator loadings are similar. For full transparency we also report that, under the Fornell-Larcker criterion, the square root of the AVE for state boredom (0.755) exceeds the latent inter-factor correlation (0.744), whereas the AVE for loneliness (0.468) is slightly below 0.50—a pattern that is acceptable when composite reliability is high (CR = 0.95 for both constructs; Fornell and Larcker, 1981; Bagozzi and Yi, 1988). We therefore conclude that, although strongly related, state boredom and loneliness are empirically distinguishable constructs in this sample.

On a practical level, these findings suggest the need for a stratified intervention strategy. For students with high levels of loneliness, simply managing stress may not be sufficient to prevent problematic viewing. Interventions must address their social connection needs simultaneously to break the stress-boredom cascade reaction. For platform designers, algorithmic systems should move beyond the logic of maximizing engagement and shift toward a digital wellbeing architecture. Specifically, for users identified as experiencing high levels of loneliness, priority should be given to promoting social connection resources rather than entertainment content.

## 5 Research limitations

The study has the following limitations. Firstly, the research was limited by the use of questionnaires, which restricts the certainty of causal conclusions. While the study based its findings on a theoretical model and suggested a directional relationship, it is possible that a reverse relationship also holds true (for example, excessive viewing might lead to increased academic stress).

Because all variables were measured concurrently, the direction of the associations cannot be determined, and alternative orderings (e.g., problematic viewing increasing academic stress, or chronic boredom heightening perceived stress) are equally compatible with the present data. The proposed mediation and moderation structure should therefore be read as a theoretically derived hypothesis to be tested with longitudinal or experimental designs.

Secondly, reliance on self-report measurement may introduce recall bias and social expectation effects, especially for sensitive behaviors such as addiction. Future research should integrate objective behavioral data (e.g., daily logs of screen time and sliding frequency indicators) and psychophysiological indicators (e.g., heart rate variability as a stress-responsiveness indicator) to verify the findings of this study.

Thirdly, the sample is limited to Chinese college students aged 18–22. Although this represents the core group of micro-drama consumers, promoting research findings to middle-aged and elderly groups or non-Chinese populations still requires caution. Cultural factors, such as the emphasis on “relationships” in collectivist cultures, may uniquely shape the experience of loneliness and its interaction with boredom in the Chinese context.

Fourth, future research can focus on intervention studies. Based on the model of this study, immediate adaptive interventions aimed at combating feelings of boredom, such as through mindfulness or attention restoration training (particularly for individuals experiencing loneliness). Finally, as AI produces an increasing number of movies and videos of ever-higher production quality, future research should examine the intrinsic factors related to addiction in AI-generated content.

Fifth, the indirect association via state boredom accounted for 15.03% of the total association between perceived stress and problematic viewing. Although indirect effects of this magnitude are typical and theoretically meaningful in behavioral-addiction research, the majority of the association remains unexplained by the present model. Critical candidate mechanisms were not measured, including trait impulsivity and self-control, fear of missing out (FoMO), escapism motives, flow experience, and platform-level affordances such as algorithmic recommendation intensity and auto-play design. In addition, the moderator and mediator were modeled at a single level; parallel or serial mechanisms (e.g., stress/loneliness/boredom/viewing) are plausible. Future studies should integrate dispositional (impulsivity, FoMO), motivational (escapism), and technological (algorithm design) factors to build a more complete explanatory model.

## 6 Conclusion

This study examined a hypothesized moderated mediation model of problematic viewing of micro-short dramas among Chinese university students. Three principal findings emerged. First, perceived stress was positively associated with problematic viewing, both directly and indirectly. Second, state boredom showed a significant indirect association linking perceived stress with problematic viewing, consistent with a partial mediation pattern: stress-related negative affective states are one pathway through which stress is connected to dysregulated media use. Third, loneliness moderated the first stage of this pathway: the association between perceived stress and state boredom, and the corresponding indirect association with problematic viewing, were significant only among students with higher levels of loneliness, supporting the characterization of loneliness as a ‘risk amplifier’ in the stress\boredom\viewing pattern.

Theoretically, these findings integrate General Strain Theory, the Functional Model of Emotion, the I-PACE model, and the vulnerability-stress framework into a single, testable account of problematic micro-short drama viewing, and they extend stress-addiction research to a new, algorithm-driven media form that has received little empirical attention despite its enormous popularity. The results also highlight the value of distinguishing state boredom—a situationally modifiable mechanism—from dispositional vulnerability factors such as loneliness, thereby specifying not only how but also for whom stress is most strongly linked to problematic viewing.

Practically, the findings support a stratified prevention logic. Because the indirect association was concentrated among high-loneliness students, loneliness screening may serve as an efficient risk marker for targeted prevention. For these students, dual-target interventions that combine stress management with structured social-connection programs are likely to be more effective than stress reduction alone. At the individual level, boredom-focused strategies—such as mindfulness training, attention training, and activity scheduling—may weaken the link between stress and viewing. At the institutional level, digital-wellbeing education that addresses algorithm-driven consumption patterns (e.g., auto-play, recommendation feeds) may help students develop more deliberate viewing habits.

Several directions for future research follow from this work. Longitudinal and experience-sampling designs are needed to establish the temporal ordering implied by the hypothesized model; experimental studies could test whether boredom induction and reduction causally shift viewing behavior. Future models should incorporate omitted mechanisms, including trait impulsivity, fear of missing out, escapism motives, flow experience, and platform-level affordances. Cross-cultural replication would clarify the generalizability of the model beyond Chinese university students, and intervention trials derived from the model would provide the strongest test of its applied value.

Given the large and growing population of university students who consume micro-short dramas daily, understanding the psychological conditions under which everyday stress is linked to problematic viewing has direct public-health relevance. We hope the present findings inform both the research agenda on emerging media addictions and the design of targeted campus mental-health programs.

## Data Availability

The datasets presented in this study can be found in online repositories. The names of the repository/repositories and accession number(s) can be found in the article/Supplementary material.
